# Enhanced Li^+^ charge storage in naphthalene diimide/vanadium pentoxide intercalates†

**DOI:** 10.1039/c8ra02970a

**Published:** 2018-07-03

**Authors:** Francisco de Araújo Silva, Renato Salviato Cicolani, Gilberto Lima, Fritz Huguenin, Grégoire Jean-François Demets

**Affiliations:** Instituto Federal de Educação Ciência e Tecnologia de São Paulo R. Américo Ambrósio 269 Sertãozinho C.E.P. 14169-263 S.P. Brazil; Departamento de Química, Faculdade de Filosofia Ciências e Letras de Ribeirão Preto, University of São Paulo Ribeirão Preto S.P. Brazil greg@usp.br +55 16 33158651 +55 16 33154860

## Abstract

*N*,*N*′-Bis(4-aminophenyl)-1,4,5,8-naphthalene diimide (NDI-ph) was intercalated into lamellar vanadium pentoxide (V_2_O_5_) in different amounts to prepare hybrid intercalates. The presence of the imide supports the material’s ability to form lithium salts with the structural stabilization of the oxide matrix. This effect is remarkable in charge/discharge cycles in terms of Li^+^ uptake and discharge by the lamellar intercalates, as we could double the ion uptake capacity (1.27 Li^+^ per V_2_O_5_ unit *vs.* 0.66 for pure V_2_O_5_), enhance the chemical reversibility and double the specific charge capacity (188 mA h g^−1^*vs.* 98 mA h g^−1^ for pure V_2_O_5_) with very small amounts of this imide. This is the first paper dealing with naphthalene diimide intercalates in vanadium pentoxide xerogel for Li^+^ storage.

## Abstract 2

*N*,*N*′-Bis(4-aminophenyl)-1,4,5,8-naphthalene diimide (NDI-ph) was intercalated into lamellar vanadium pentoxide (V_2_O_5_) in different amounts to prepare hybrid intercalates.

## Graphical abstract

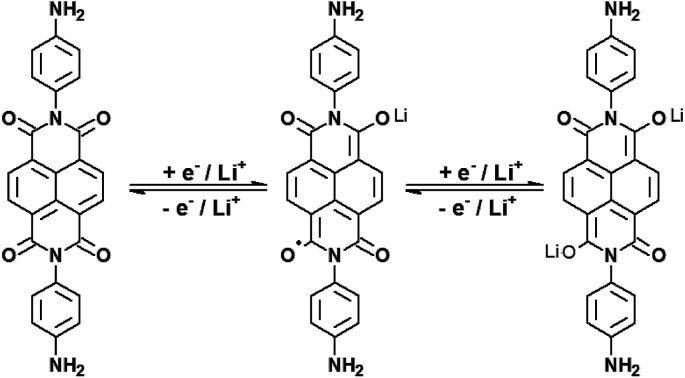

## 1 Introduction

Vanadium pentoxide is probably the most popular lamellar conducting oxide ever reported in the literature.^1^ Since the pioneering studies of Livage^2–5^ and Oka^6^ in the 80s, thousands of papers have been published exploring its ability to include cations reversibly during the reduction process.^7–10^ This property has been explored in most of the papers dealing with it, in the form of using V_2_O_5_·*n*H_2_O xerogels (VXG) or nanostructures to develop cathodic materials for Li^+^ ion batteries in several studies with modified electrodes. In VXG, V^5+^ ions are connected to each other through oxo-bridges, and are surmounted by apical double-bonded oxygen atoms forming pyramidal-like monomeric units, which polymerize in ribbons and sheets separated by counter-ions and water molecules. Vanadium(v) centers may be reduced to vanadium(iv) chemically or electrochemically, and the presence of both in the structure is responsible for phenomena such as intervalence electron transitions, electron-hopping conduction and electrochromism.^11–13^

Electroinsertion occurs when V^5+^ ions are electrochemically reduced to V^4+^, and the oxide admits a cation between its lamellae to compensate for the charge, as shown in eqn (1)

1 V^5+^ + e^−^ ↔ V^4+^ + Li^+^

**Fig. 1:**
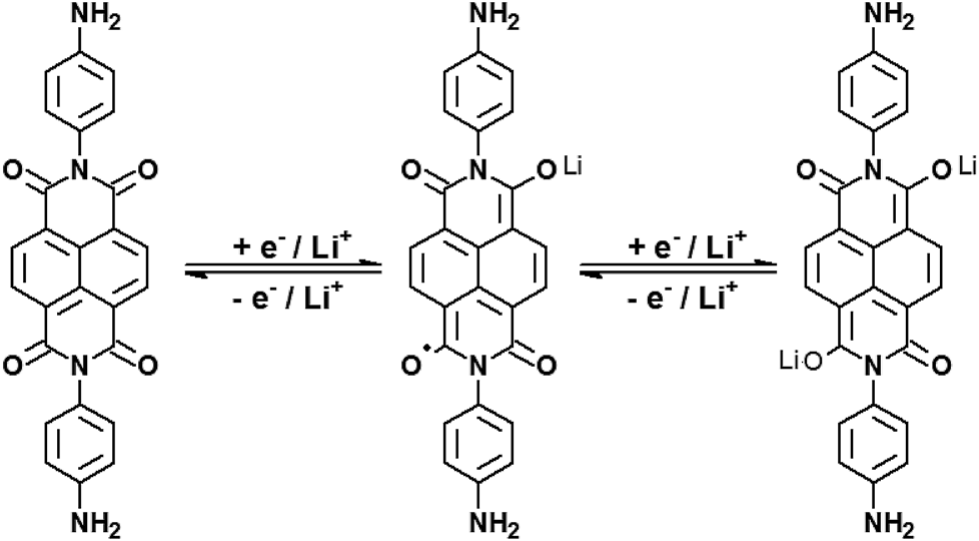
Successive electrochemical reductions of *N*,*N*′-bis(4-aminophenyl)-1,4,5,8-naphthalene diimide, or NDI-ph, in the presence of lithium ions.

Despite the simplicity it offers for building batteries, V_2_O_5_·*n*H_2_O has several experimental limitations. The most important is the existence of multiple intercalation sites that can be more or less reversible towards Li^+^ ion intercalation. This major drawback reduces its charge storage capacity with time, and also generates structural stress due to charge trapping that is slowly released *via* structural modification. In other words, the theoretical capacity of 294 mA h g^−1^ (2 electrons per V_2_O_5_ unit)^14^ is hardly observed in the literature in common setups (organic solvents and LiClO_4_ electrolytes for example) and its performance usually falls dramatically after 20 or 30 charge/discharge cycles. A way to avoid such degradation is by using intercalated auxiliary species, electroactive or not, in the form of nanocomposites, to hold the structure and to facilitate Li^+^ flux in the interlamellar space. This strategy usually increases the charge capacity of the composites and their cyclability when compared to pure V_2_O_5_ matrices. Naphthalic diimides are interesting molecules for this purpose for many reasons: they are easy to prepare, they are very resistant to hard chemical environments, and most importantly, they are electron acceptors. Naphthalimide cores may undergo two successive reductions, generating very stable radical anions and dianions (Fig. 1).^15^

Their rigid planar structure will allow for the preparation of intercalates with VXG, and their reduction inside the lamellar space of the oxide will contribute to Li^+^ uptake during the reduction of the whole matrix at least by connecting redox sites through the matrix. Their presence should stabilize the matrix too, as it will reduce the breathing of the oxide structure by the host–guest electrostatic interactions. Furthermore, we may enhance the host–guest interactions using aromatic and/or hydrogen-binder radicals and substituents in the naphthalene diimide.^16^ Recently, a few papers^16–24^ have demonstrated that these imides may act as cathode materials for lithium batteries but none, to the best of our knowledge, describe naphthalimide/oxide composites for this purpose. We have prepared in the present paper a series of VXG intercalates with *N*,*N*′-bis(4-aminophenyl)-1,4,5,8-naphthalene diimide (from now on referred to as NDI-ph) over fluorine-doped conductive glass electrodes. NDI-ph was chosen because it can be modified with aromatic groups and because it has amines that may bind water or the oxide in the interlamellar gap and stabilize the structure. The intercalation of this imide doubles the charge capacity of VXG and enhances its lithium ion uptake.

## 2 Experimental details

NDI-ph was prepared following the general procedure described in the literature:^16,25–27^ 1 mmol of 1,4,5,8-naphthalenetetracarboxylic dianhydride (268 mg) was mixed with 10 mmol of 1,4-phenylenediamine (1.05 g) and 20 mL of dimethylformamide under reflux for 8 h. The solvent was removed at reduced pressure and the solid was washed with water, and recrystallized in DMF. It was washed a second time with water and dried in a desiccator for 3 hours, and in an oven at 90 °C for one hour. Yield = 82% (^1^H-NMR, FTIR, mass specs. and UV-vis spectra are available in the ESI in Fig. S1–S4†). V_2_O_5_·*n*H_2_O was obtained *via* the sol–gel route described elsewhere,^28–31^ starting from a 0.1 mol dm^−3^ NH_4_VO_3_ solution. The solution was passed through a 50W-2x Dowex cationic exchange resin column (pH = 4) to form HVO_3_, which was then left to polymerize for a week.

With 50-day-old gels,^32^ we have prepared 3 intercalates containing NDI-ph in different amounts: 1 mg NDI-ph in 6 cm^3^ VXG (VXG/NDI-ph1), 3 mg in 6 cm^3^ (VXG/NDI-ph3) and 6 mg in 8 cm^3^ (VXG/NDI-ph6), resulting in concentrations of 0.17 mg mL^−1^, 0.5 mg mL^−1^ and 0.75 mg mL^−1^ (Fig. S5†). Using higher quantities of imide destabilizes the gels. The 3 mixtures, as well as a pure 6 cm^3^ gel control sample, were left under stirring for 48 h. To avoid the formation of cloths, all the samples were subjected to an ultrasound bath for 10 min every 12 hours. The pure gel (VXG) and the three samples (VXG/NDI-ph1, VXG/NDI-ph3 and VXG/NDI-ph6) were dried at room temperature, leading to homogeneous orange xerogels.

FTIR spectra were obtained on an ABB Bomem MB 100 spectrophotometer using KBr pellets. ^1^H-NMR spectra were measured on a Bruker Advance DRX 500 500 MHz spectrometer in DMSO-*d*6 (ESI†). UV-vis spectra were obtained on a Shimadzu UV 1280 spectrophotometer using 1 cm optical path quartz cuvettes. Mass spectra were obtained using a MALDI-TOF/TOF Ultraflextreme Bruker Daltonics instrument using a 2,5-dihydroxylbenzoic acid (DHB) matrix. Electron microscope images were obtained on a Zeiss EVO 50 SEM microscope. X-ray diffraction analysis (PXRD) was carried out using a Siemens 5005 diffractometer equipped with a copper lamp (*λ* = 1.54 Å), 2*θ* from 2° to 50°, 0.05° s^−1^. Thermogravimetric analysis was performed on TA Instruments – Q600 SDT 2960 DSC-TGA equipment (air, heating rate 10 °C min^−1^). AFM images were obtained on a Shimadzu SPM 9600 microscope in dynamic mode. Electrochemical measurements were made on a μAutolab III potentiostat using a conventional three electrode arrangement, with a Pt auxiliary electrode and an Ag^+^/AgNO_3_ 0.01 mol dm^−3^ in acetonitrile reference electrode. The working electrodes were fluorine-doped tin oxide electrodes (FTO, *R* < 20 Ω) modified by dip-coating (single immersion) with pure VXG or the intercalate xerogels. The measurements were made in acetonitrile/LiClO_4_ 0.1 mol dm^−3^. Chronopotentiometry measurements were normalized with regards to the area of the films (1 cm^2^) and the V_2_O_5_ content, which was measured using spectrophotometry according to the procedure described elsewhere.^31,33,34^ Electrochemical impedance spectra were obtained from 10 kHz to 0.1 Hz, using a 5 mV ac amplitude.

## 3 Results

**Fig. 2:**
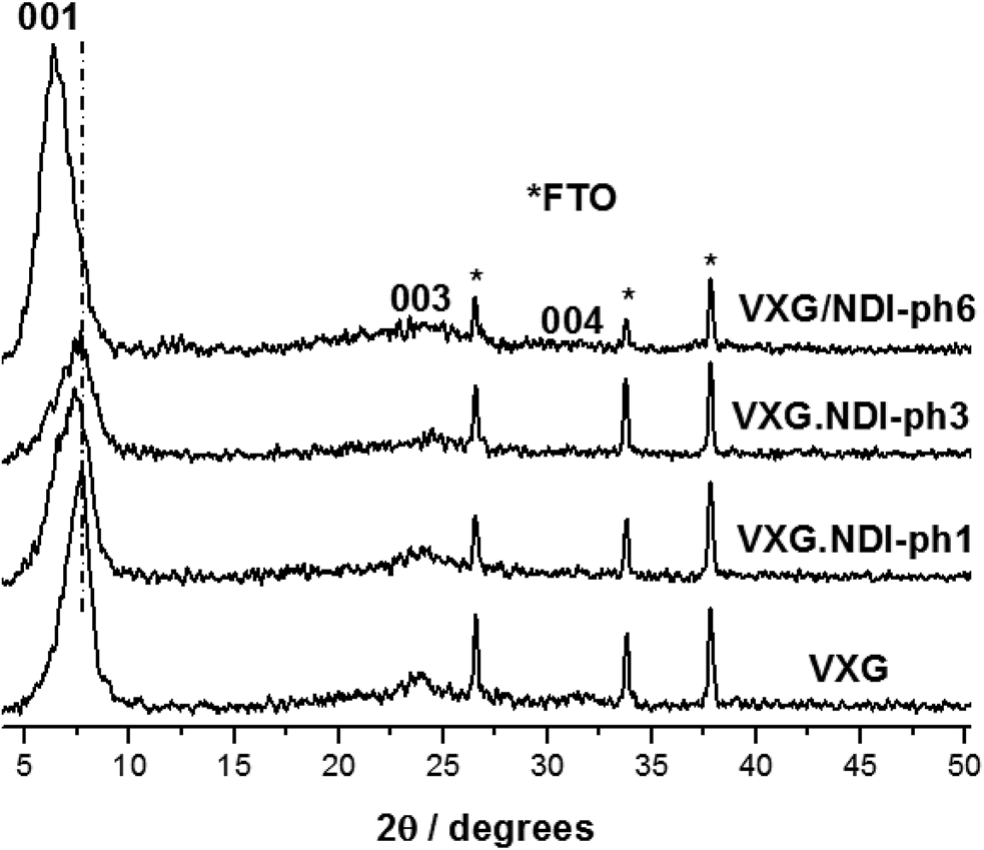
XRD patterns of VXG and the VXG/NDI-ph (1, 3 and 6) intercalates (*λ* Cu Kα = 1.54 Å).

The formation of lamellar intercalates rather than simple mixtures is clear from the XRPD patterns (Fig. 2), which display increasing basal distances ranging from 11.42 Å to 11.92 Å for VXG/NDI-ph1, 12.02 Å for VXG/NDI-ph3 and 13.76 Å for VXG/NDI-ph6. These distances indicate that the imide moieties lie parallel to the oxide sheets rather than inclined, like other diamines, since NDI-ph is 28 Å long and non-coplanar arrangements would increase the basal distance much more than this, even at low concentrations.^4–6,12,31,32,35–39^

Their approximate formulae were calculated from thermogravimetric data as V_2_O_5_·(H_2_O)_2.1_, for pure xerogel (control), V_2_O_5_·(NDI-ph)_0.017_(H_2_O)_2.2_ for the sample VXG/NDI-ph1, V_2_O_5_·(NDI-ph)_0.026_(H_2_O)_2.2_ for VXG/NDI-ph3 and V_2_O_5_·(NDI-ph)_0.032_(H_2_O)_2.2_ for the VXG/NDI-ph6 intercalate, assuming that the crystal water amount was almost constant for all samples (Fig. S6†). The FTIR spectra show both the imide and the oxide bands, and the former get more intense as the amount of imide increases in the samples. V_2_O_5_ bands are present at 509 cm^−1^ (sym. *ν* V–O–V) and 762 cm^−1^ (antisym *ν* V–O–V), and there is also a V=O stretching band at 1010 cm^−1^. The imide bands appear at 1351 cm^−1^ and 1337 cm^−1^ (assigned to the Ar-H stretching modes) as well as 1670 cm^−1^ and 1715 cm^−1^ (assigned to the carbonyl group stretching modes) (Fig. S7†).^12,31,37–40^

**Fig. 3:**
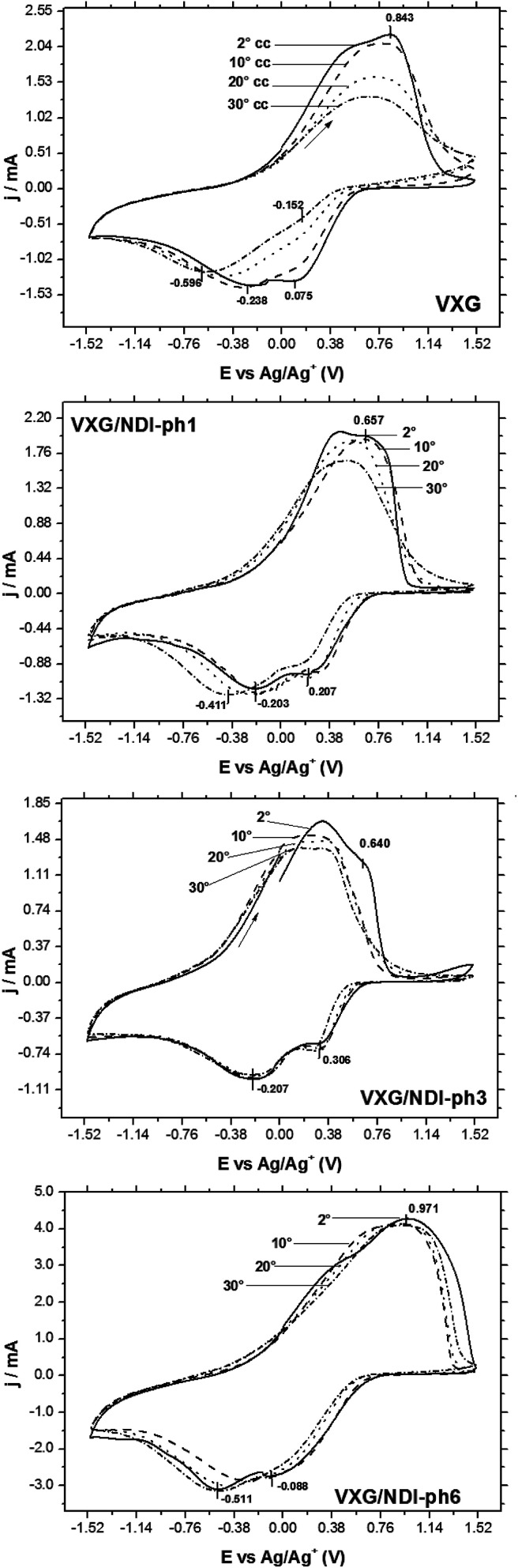
Voltammograms of VXG and VXG-ph (1, 3 and 6) after 2, 10 and 30 cycles at 100 mV s^−1^.

All of the samples display the typical voltammetric behavior of vanadium-pentoxide-modified electrodes during electrochemical cycles (Fig. 3). The voltammograms evolve over time and change in shape, and this is associated with the electrochemical conditioning process of the oxide when structural stress is released *via* Li^+^ ion insertion and solvent rearrangements in the intercalation matrix. In the case of the pure oxide, two pairs of voltammetric waves tend to merge into a single one during successive cycles. This process is known as the conditioning of the matrix, and it reveals a uniformization of the redox centers in the matrix after structural rearrangement and solvent and charge balancing.^41^ Two reduction processes at 0.075 V and −0.238 V *vs.* Ag/Ag^+^ and a superimposed oxidation process at 0.843 V are shown. These reduction processes correspond to two different types of Li^+^ intercalation site, and both merge after 30 cycles into a single signal at −0.596 V, which is related to structural stress relief. The voltammograms of the intercalates display a different behavior, and both intercalation sites seem to be maintained and accessible for all of the electrodes, even after 30 potential cycles. As the amount of diimide increases, the voltammograms seem to vary less than they do for neat VXG, and more than 2 processes are visible, especially when cycling at sweep rates as slow as 20 mV s^−1^ (Fig. S9–S11†). Other authors have assigned this behavior to the decrease of crystallinity due to the presence of the guest molecules^42^ and, due to its low concentration, it could not be assigned to the oxidation and reduction of the organic moiety in the interlamellar space, or to the creation of new Li^+^ allocation sites alongside the NDI-ph molecules in the bulk materials.

**Fig. 4:**
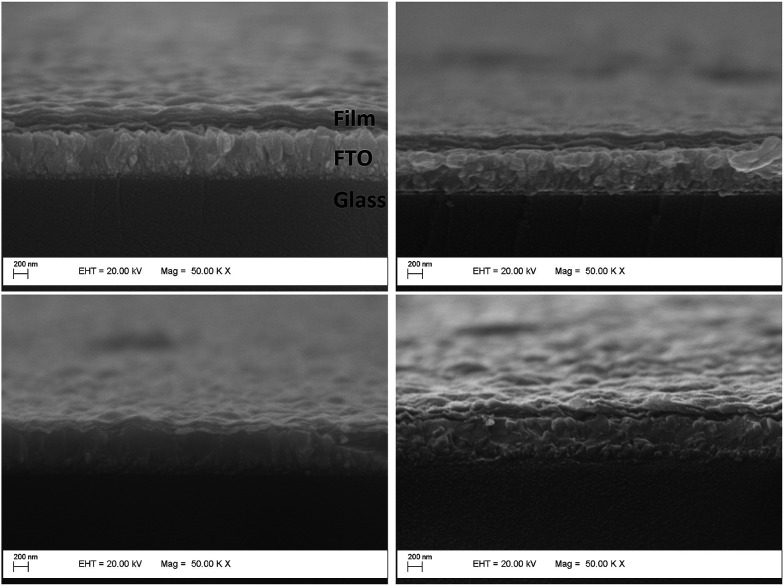
Electron microscope images of the modified FTO electrodes, showing the composite layer regularity and adhesion to the electrode surface.

In order to estimate the surface effects in the electrochemical results, we have used AFM imaging (ESI†). All of the films have similar rugosities, meaning that the areas exposed to the electrolyte per electrode area (5 μm^2^) are similar (6.437 μm^2^ for VXG, 6.437 μm^2^ for VXG/NDI-ph1, 6.447 μm^2^ for VXG/NDI-ph3 and 6.483 μm^2^ for VXG/NDI-ph6) (Fig. S8†). The thickness of the intercalate films (around 200 nm) is smaller than that of the VXG film (around 300 nm), as can be seen in the SEM images (Fig. 4).

**Fig. 5:**
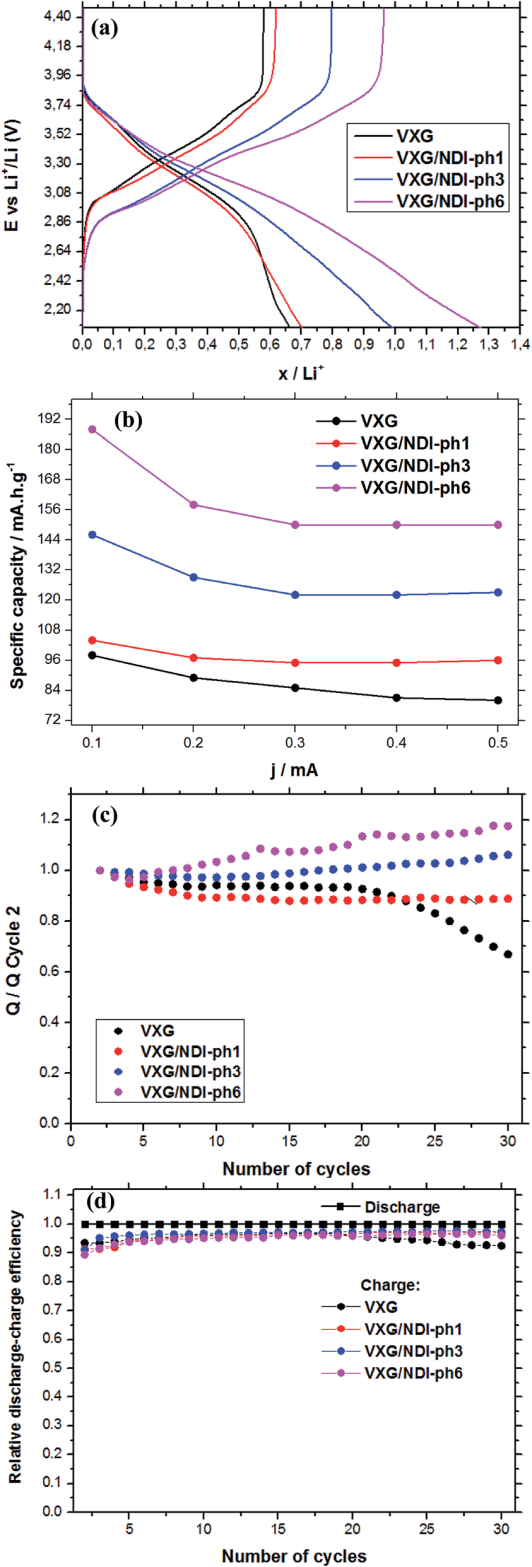
(a) The amount of lithium ions inserted into the films as a function of the discharge/charge current from chronopotentiometry (cutoff *E* 4.47 V to 2.07 *vs.* Li/Li^+^, 0.2 mA) per mol of V_2_O_5_; (b) the specific charge capacity at various current densities; (c) the discharge variation during 30 electrochemical cycles, compared to the second cycle (*Q*_*n*_/*Q*_2_, at 0.2 mA); (d) coulombic efficiency during 30 charge/discharge cycles.

Fig. 5a shows the amount of lithium ions inserted into the films during potential sweeps from 1.2 V to −1.2 V (*vs.* Ag/Ag^+^) with a current density of 0.1 mA per mol of V_2_O_5_. By considering the mass of vanadium and the raw formulae of the composites, we can calculate their formulae after lithium ion insertion as: Li_0.66_V_2_O_5_(H_2_O)_2.2_ for the control sample, and Li_0.70_(NDI-ph)_0.017_V_2_O_5_(H_2_O)_2.2_, Li_0.99_(NDI-ph)_0.026_V_2_O_5_(H_2_O)_2.2_ and Li_1.27_(NDI-ph)_0.032_V_2_O_5_(H_2_O)_2.2_ for VXG/NDI-ph1, VXG/NDI-ph3 and VXG/NDI-ph6, respectively. Lithium insertion into the intercalates is linearly proportional to the NDI-ph content. Fig. 5b shows their specific charge capacity in mA h g^−1^ (1.2 V to −1.2 V at 0.1, 0.2, 0.3, 0.4 and 0.5 mA), normalized *via* the vanadium mass in the films. As the current decreases, the specific charge capacity increases as expected for this kind of system. This is related to the diffusional control at higher currents. This was also observed in the cyclic voltammetry experiments at different sweeping rates (ESI†), since redox peak separation is very dependent on sweeping rate.

In Fig. 5a we can observe clearly the dependence of the amount of lithium ions inserted per V_2_O_5_ units in the films on the NDI-ph concentration. When examining the specific charge capacity curves of the intercalates, a gradual increase from 0.65 Li^+^ to 0.7, 1.0 and 1.25 is observed for the intercalates compared to neat VXG films. The value for the VXG/NDI-ph6 intercalate is twice that of the pure VXG films. With a 0.1 mA current density, the specific charge capacity values reached 98 mA h g^−1^ for pure VXG, 104 mA h g^−1^ for VXG/NDI-ph1, 146 mA h g^−1^ for VXG/NDI-ph3, and 188 mA h g^−1^ for VXG/NDI-ph6, which represent a dramatic increase in the values in the presence of NDI-ph.

**Fig. 6:**
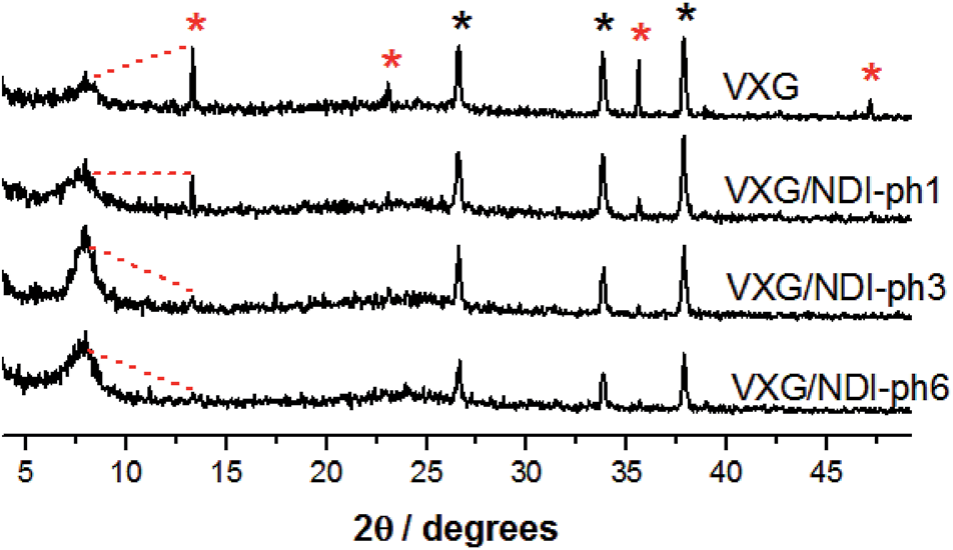
XRD patterns of VXG and the VXG/NDI-ph (1, 3 and 6) intercalates (*λ* Cu Kα = 1.54 Å) after 30 electrochemical cycles (cutoff *E* 4.47 V to 2.07 *vs.* Li/Li^+^, *j* = 0.2 mA). The red asterisks belong to the lithium-bearing phase, and the black asterisks belong to the fluorine-doped thin oxide of the electrodes.

During the first 30 charge–discharge cycles, when compared to the second cycle with a current of 0.2 mA, we can see that the Li^+^ insertion capacity of the NDI-ph-bearing films is always higher than it is in the absence of NDI-ph, since all of the composites displayed higher cyclabilities than pure VXG did. One can see that the electrochemical process becomes progressively more reversible (the lithium insertion and deinsertion curves at this current density are available in the ESI in Fig. S12†). This is clear evidence that the imide provides a more stable structural organization in the matrix towards electrochemical cycles, facilitating Li^+^ flux in the interlamellar gap, which is related to their capacity to form salts as mentioned above (Fig. 1). XRPD measurements were carried out after 30 electrochemical cycles for all of the composites (Fig. 6). It is clear that the lamellar structure was preserved as the amount of diimide increased in the samples. Furthermore, its presence probably prevents the formation of new lithium-bearing phases such as LiV_3_O_8_.^43,44^

Fig. 5d shows a comparison of the coulombic efficiency of the films. In the figure, the amount of Li^+^ ions that enter the film (the cathodic efficiency) is normalized to 1 (100%) and the anodic efficiency (the charge process, or the amount of Li^+^ ions leaving the films) is plotted against this value. For all the films, the amount of Li^+^ ions leaving the films is always lower than 1, indicating that a small amount of ions get stuck in the structure, but this amount varies very little for the NDI-ph intercalates, and their efficiency always remains above 97%. In pure VXG however, the reversibility hardly reaches 93%, and this number decreases rapidly after only 20 cycles.

**Fig. 7:**
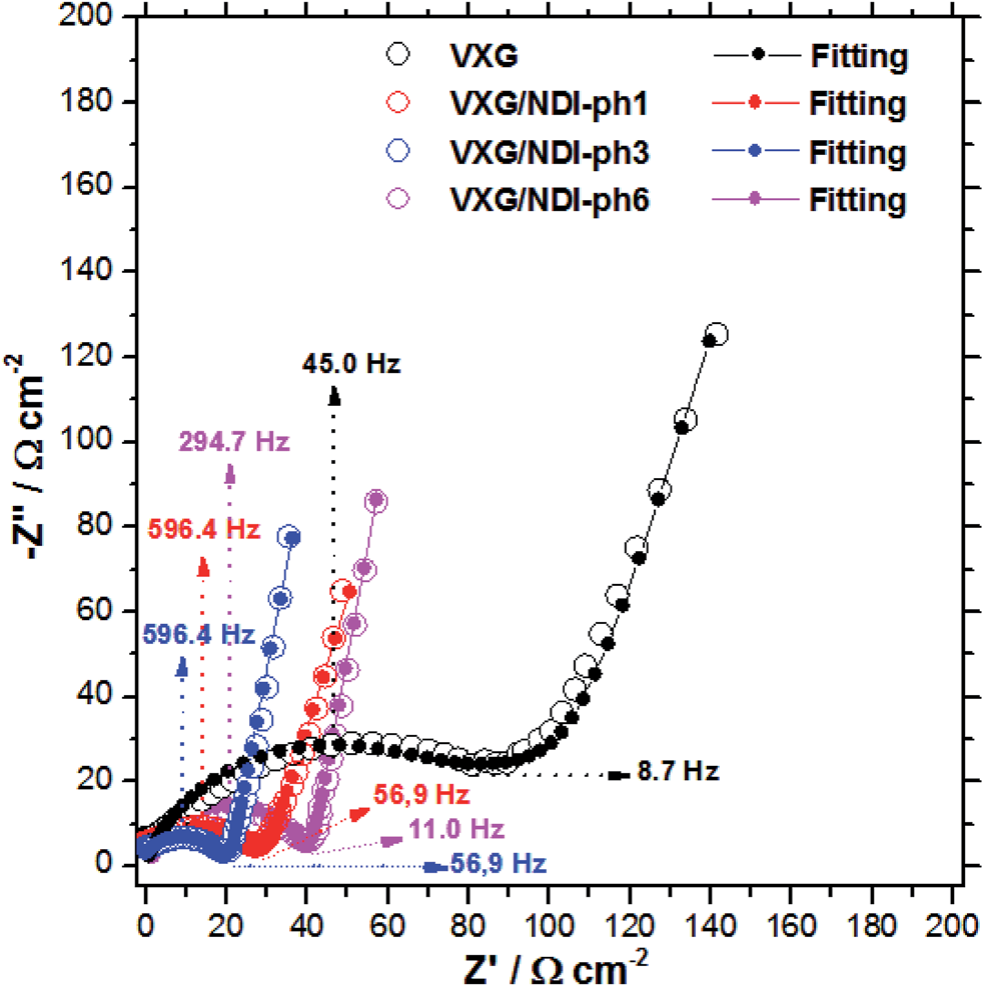
Experimental and adjusted impedance spectra for lithium ion electro-insertion in VXG (black), VXG/NDI-ph1 (red), VXG/NDI-ph3 (blue) and VXG/NDI-ph1 (magenta) at 0.3 V. *R*_Ω_ was subtracted for better comparison; these values are reported in Table 1.

Electrochemical impedance spectroscopy (EIS) measurements provided new insights into the lithium ion electro-insertion into the VXG-ph (1, 3 and 6) intercalates. The impedance of the lithium ion electro-insertion can be expressed as a function of the double-layer capacitance (*C*_dl_), the charge transfer resistance (*R*_ct_) and the diffusion impedance (*Z*_d_), and can be modelled according to eqn (2) and (3),^45,46^2
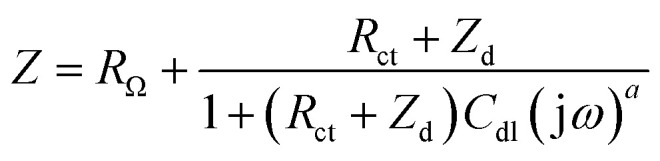
where *R*_Ω_ corresponds to the sum of the bulk, the electrolytic solution and the contact resistance, *ω* is the angular frequency, *α* is a dimensionless parameter and j is the imaginary unit. The model of the anomalous diffusion due to frequency dispersions was used to represent the diffusion impedance (eqn (3)),^47^3
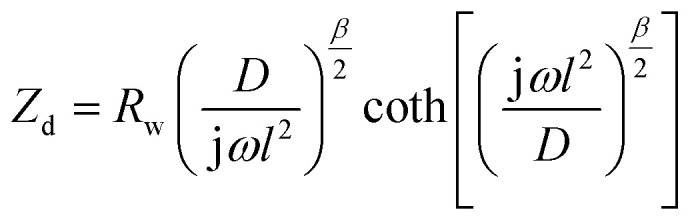
where *R*_w_ is the diffusion resistance, *D* is the diffusion coefficient, *l* is the host matrix thickness (the length of the diffusion pathway) and *β* is a dimensionless parameter. Fig. 7 shows the Nyquist diagrams for the VXG and VXG-ph (1, 3 and 6) modified electrodes at 0.3 V and Table 1 summarizes the electrochemical parameter values obtained from the impedance data fitting.

**Table 1:** E.I.S. adjustment data

Sample	*R*_Ω_ (Ω cm^2^)	*R*_w_ (Ω cm^2^)	*R*_ct_ (Ω cm^2^)	*C*_dl_ (μF cm^−2^)	*D*/*l*^2^ (s^−1^)	*D* (10^−9^ cm^2^ s^−1^)	*α*	*β*	Error (%)
VXG	43.3	90	75.3	90	2.00	1.82	0.75	0.81	1.84
VXG/NDI-ph1	31.0	7.4	29	90	20.20	10.3	0.70	0.80	1.65
VXG/NDI-ph3	26.0	6.2	21.3	85	23.50	4.90	0.72	0.88	1.30
VXG/NDI-ph6	35.5	4.66	39	68	36.12	14.4	0.79	0.88	0.0080

At high frequencies in the Nyquist diagram, the semicircle observed refers to the charge transfer resistance at the electrode–electrolyte interface in parallel with an electrical double layer capacitance. The modified electrodes with VXG/NDI-ph1, ph3, and ph6 exhibited lower *R*_ct_ values (associated with the Li^+^ electro-insertion) than the neat VXG electrode did, indicating a faster lithium electro-insertion from the solution to the modified host matrices. This reduction cannot be assigned to the surface effect, according to the AFM data, and it is a clear indication that the imides play an important role by lowering the potential barrier at the interface.

For pure VXG, it is possible to observe a discrete transition region from a semi-infinite to finite diffusion regime at lower frequencies, but this transition is absent on the intercalate spectra, which display only a finite diffusion regime. In other words, the composites show higher ionic mobilities than the neat vanadium pentoxide does.

*l* values for the VXG and VXG-ph (1, 3 and 6) electrodes correspond to 302, 226, 144 and 200 nm, respectively, and the adjusted *D*/*l*^2^ values allow us to determine the diffusion coefficient in the films (Table 1). The *D* values determined for the VXG modified with NDI-ph were ten times higher than those for neat VXG, indicating a faster lithium ion diffusion rate. The diffusion resistance, *R*_w_, for the VXG electrode was also higher than for the other intercalates, suggesting that the intercalation processes in V_2_O_5_ are influenced by diffusion as well as by *R*_ct_. In the composites, the *R*_w_ values were significantly lower, indicating that the imides facilitate ionic transit. These impedance data agree with the electrochemical results in the time domain shown in the cyclic voltammetry, which indicates a low practical irreversibility.

## 4 Conclusions

We prepared intercalates of *N*,*N*′-bis(4-aminophenyl)-1,4,5,8-naphthalene diimide in lamellar vanadium pentoxide, and these hybrid materials were able to ally the properties of the oxide to those of the imides to prepare high-performance matrices for the electro-insertion of Li^+^ ions for batteries for example. The diimides undergo two successive reductions that enhance the lithium ion uptake by the metal oxide during electrochemical reduction. Furthermore, they act as structural binders, preventing the oxide structure from collapsing after successive electrochemical cycles. This effect is remarkable in charge/discharge cycles in terms of Li^+^ uptake and discharge by the lamellar intercalates, as we could double the ion uptake capacity (1.27 Li^+^ per V_2_O_5_ unit *vs.* 0.66 for pure V_2_O_5_), considerably enhance the chemical reversibility and double the specific charge capacity (188 mA h g^−1^*vs.* 98 mA h g^−1^ for pure V_2_O_5_) with very small amounts of this imide. Even in small amounts, such imides seem to provide alternative paths for charge carriers, by connecting electrochemical redox centers and enhancing the access to electroactive sites. Other imides act in a similar way, and we have prepared a series of them. A systematic study with different substituents on the imide cores will be the subject of another paper.

## Conflicts of interest

There are no conflicts to declare.

## Supplementary Material

RA-008-C8RA02970A-s001
