# Supplementary material for: Enhanced Li+ charge storage in naphthalene diimide/vanadium pentoxide intercalates
Source: RSC Adv. 2018 Jul 3;8(42):24029–35. doi: 10.1039/c8ra02970a (PMC9081847; doi:10.1039/c8ra02970a)
Supplement: RA-008-C8RA02970A-s001 [file RA-008-C8RA02970A-s001.pdf]

Electronic Supplementary Material (ESI) for RSC Advances.
This journal is © The Royal Society of Chemistry 2018

Electronic supplementary information for : Enhanced Li^+ Charge Storage in Naphthalene Diimide/Vanadium Pentoxide Intercalates.

Francisco de Araújo Silva,[a] Renato Salviato Cicolani,[a], Gilberto Lima[a], Fritz Huguenin and Grgoire Jean-François Demets* [a]

Received Xth XXXXXXXXXX 2013, Accepted Xth XXXXXXXXX 201X
First published on the web Xth XXXXXXXXXX 201X
DOI: 10.1039/b000000x

1 NDI-ph caracterization

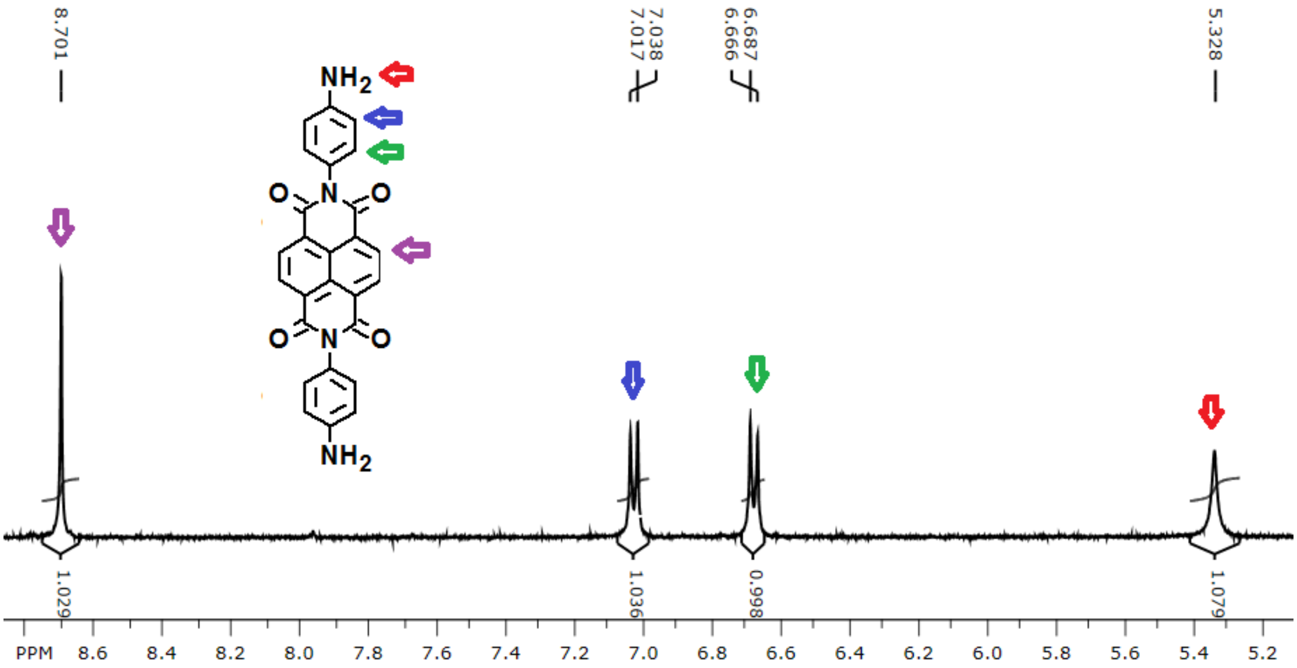

Fig. 1 ^{1}H-NMR spectrum of NDI-ph.

[a]*Laboratório de Materiais e Interfaces Moleculares, DQ-FFCLRP Universidade de São Paulo. Av Bandeirantes 3900 CEP 14040-901; Ribeirão Preto, S.P., Brazil. Fax: 5516 36024861; Tel: 5516 36024860; E-mail: greg@usp.br*

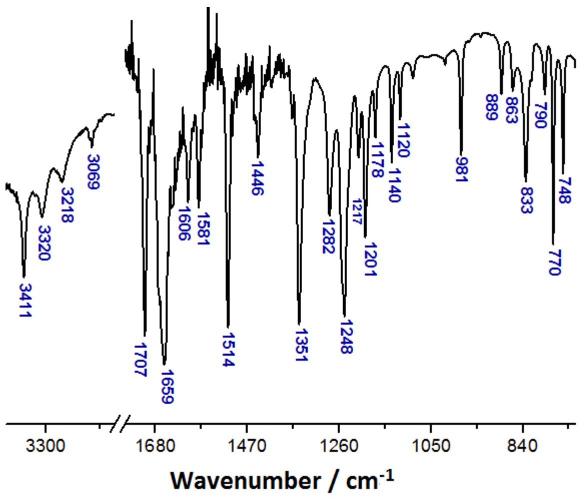

Fig. 2 FTIR spectrum of NDI-ph.

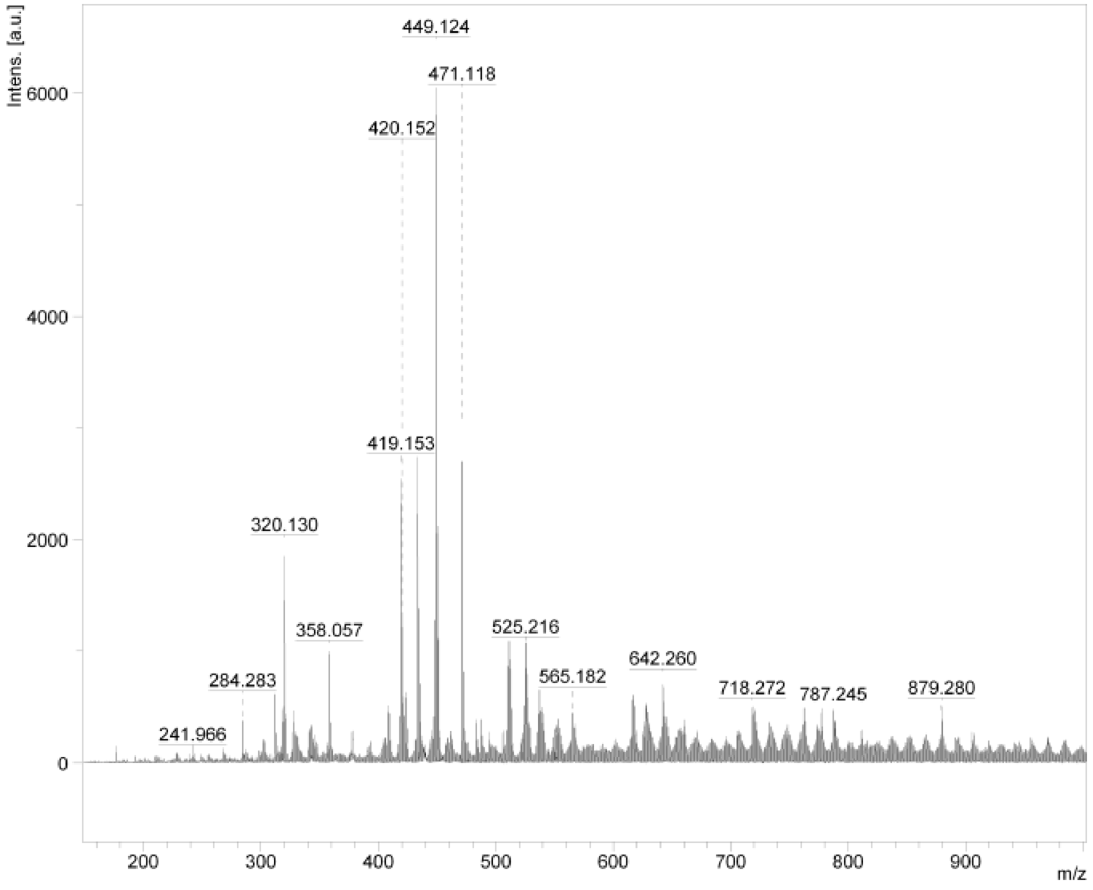

Fig. 3 Maldi-TOF for NDI-ph.

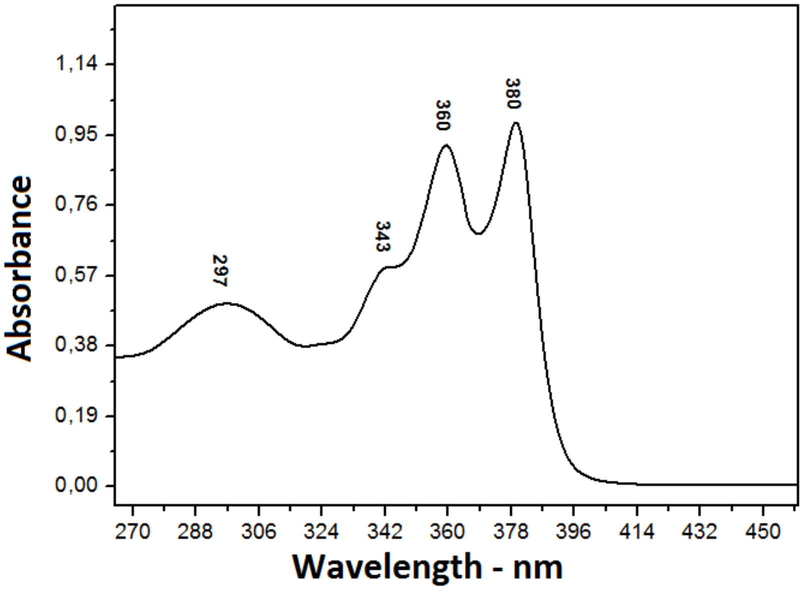

Fig. 4 Electronic spectrum of NDI-ph in DMF

2 Composites caracterization

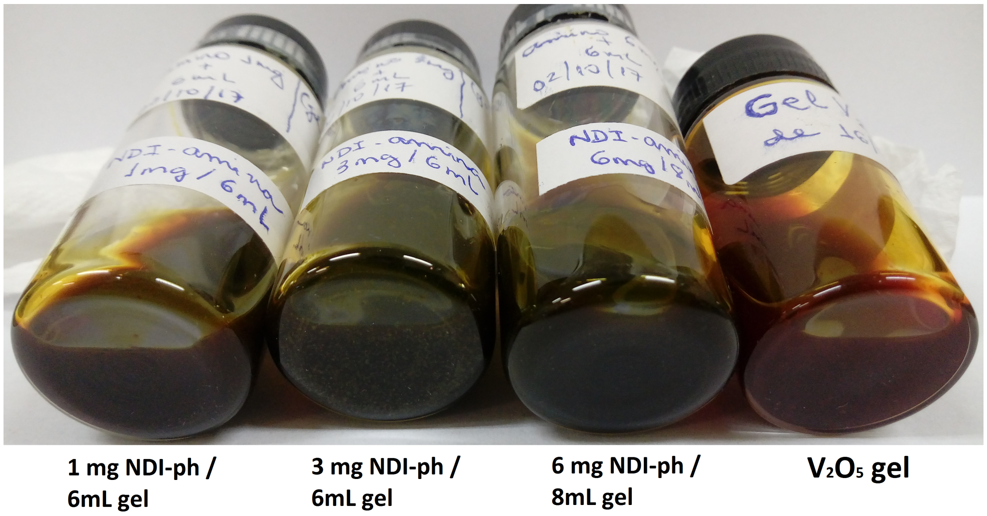

Fig. 5 Gels containing NDI-ph, and the control sample, before drying.

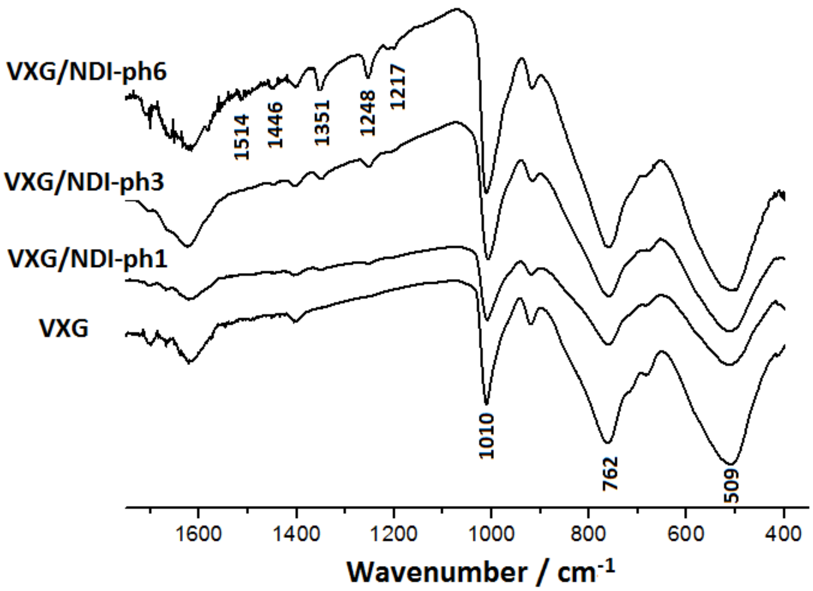

Fig. 6 FTIR spectra of the composites and VXG.

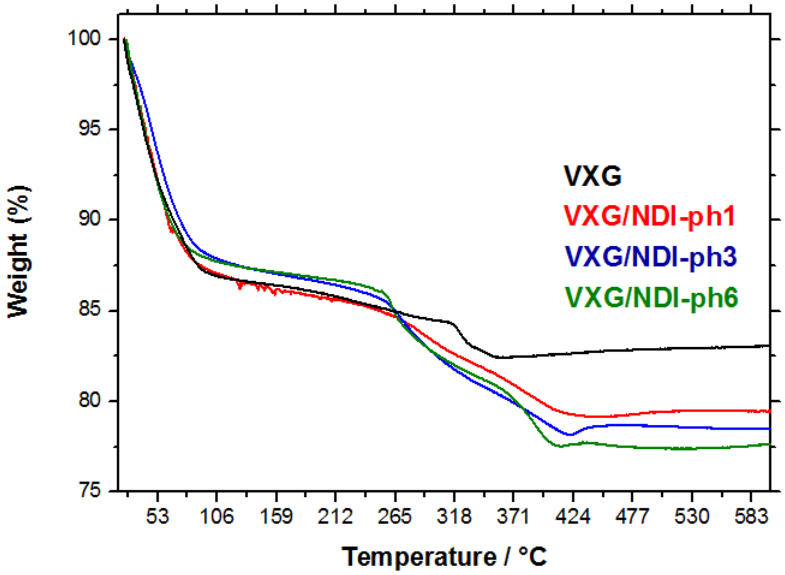

Fig. 7 Thermogravimetric analysis of the composites and VXG.

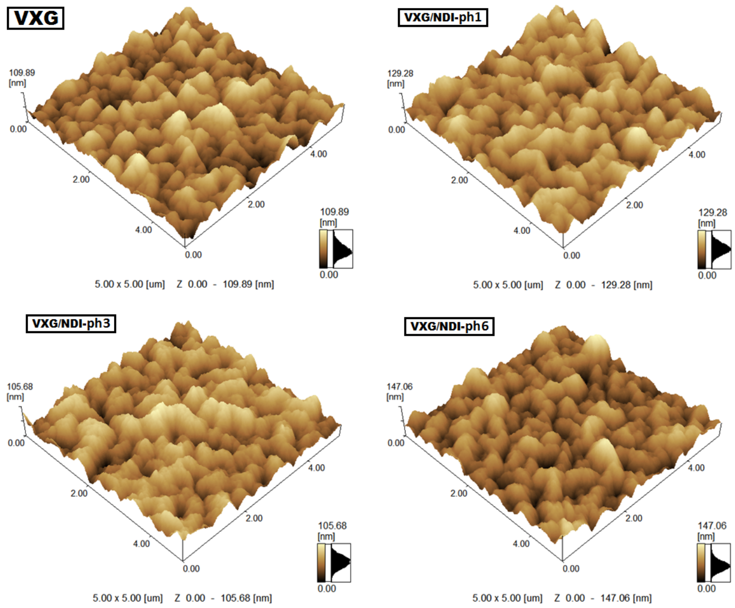

Fig. 8 afm

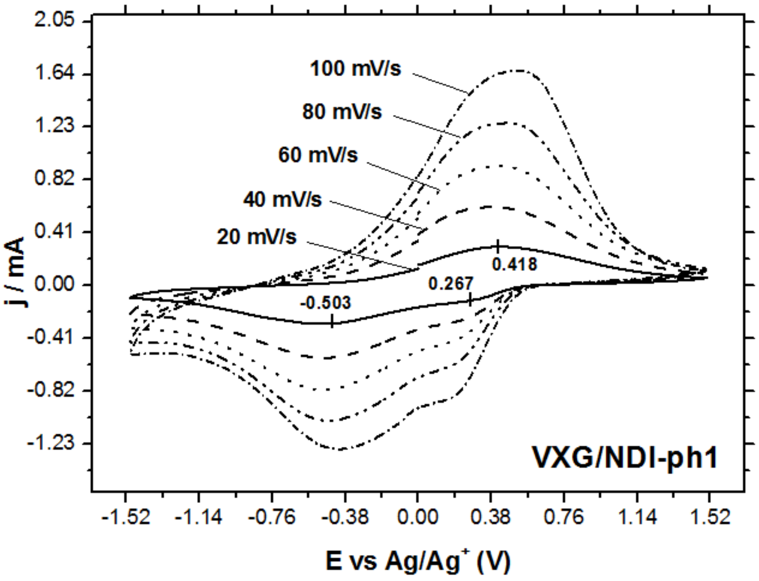

Fig. 9 Cyclic voltammograms of VXG. at 20mV/s ($LiClO_4$/MeCN).

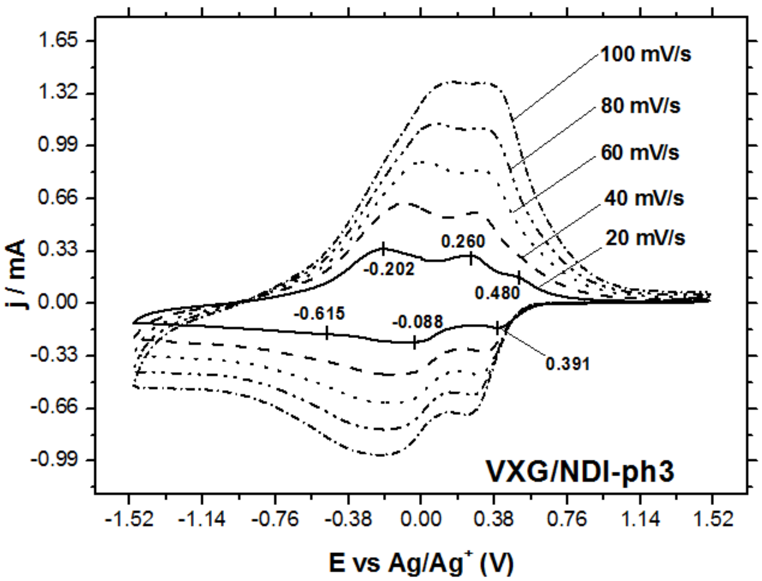

Fig. 10 Cyclic voltammograms of VXG at 20mV/s ($LiClO_4$/MeCN).

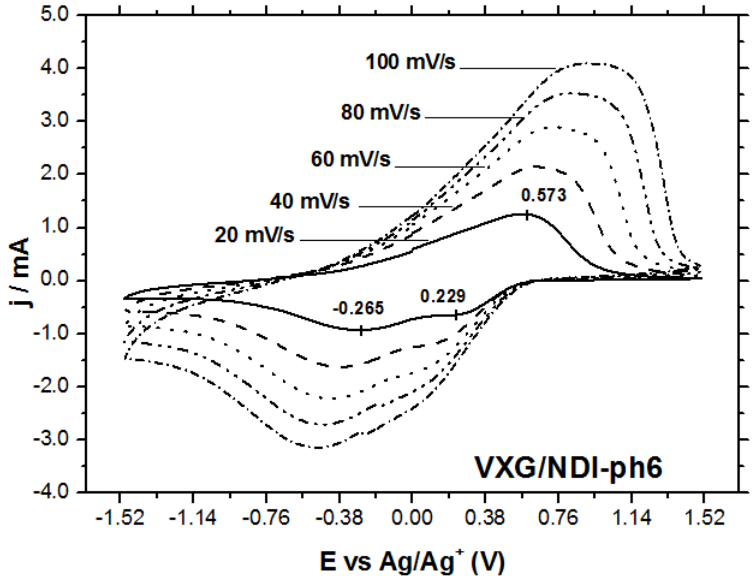

Fig. 11 Cyclic voltammograms of VXG.at 20mV/s ($LiClO_4$/MeCN).

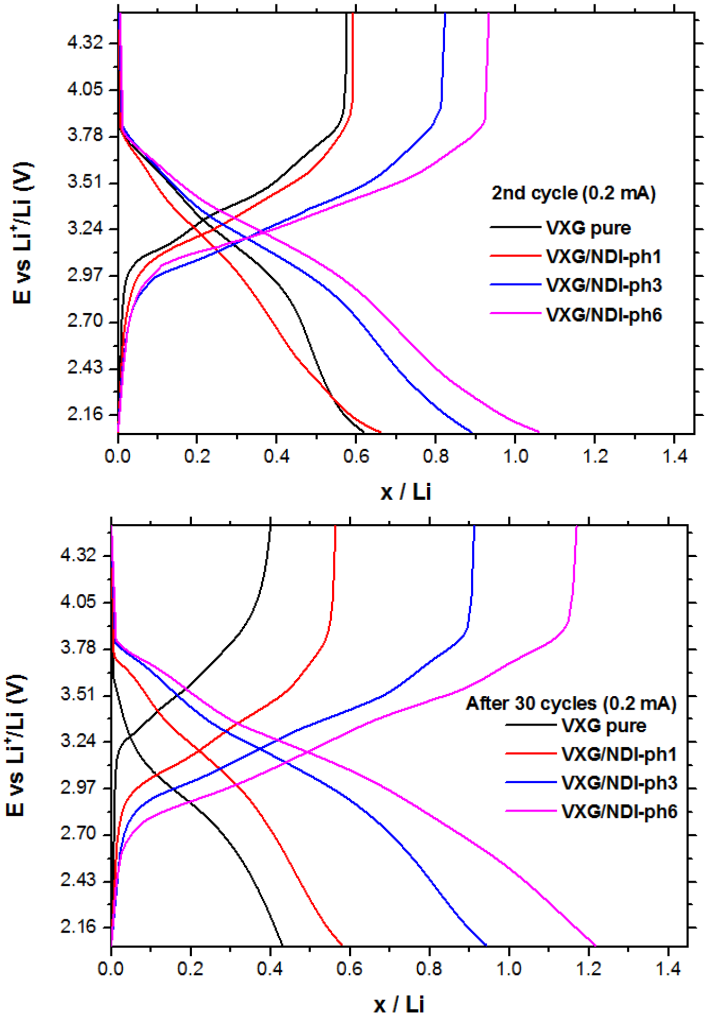

Fig. 12 (a) Lithium ion inserted/extracted in the films as a function of discharge/charge current (cutoff E 4.47 V to 2.07 vs Li^+/Li, j = 0.2 mA) per mol of V_2O_5; (b) Lithium ion inserted/extracted in the films as a function of discharge/charge current (cutoff E 4.47 V to 2.07 vs Li^+/Li, j = 0.2 mA) per mol of V_2O_5 - after 30th cycles.
